# The long noncoding RNA SPRY4-IT1 increases the proliferation of human breast cancer cells by upregulating ZNF703 expression

**DOI:** 10.1186/s12943-015-0318-0

**Published:** 2015-02-22

**Authors:** Yongguo Shi, Juan Li, Yangchen Liu, Jie Ding, Yingrui Fan, Yun Tian, Li Wang, Yifan Lian, Keming Wang, Yongqian Shu

**Affiliations:** Department of Oncology, The Second Affiliated Hospital of Nanjing Medical University, Nanjing, Jiangsu PR China; Taixing People’s Hospital, Taixing, Jiangsu PR China; Department of Oncology, the First Affiliated Hospital of Nanjing Medical University, Nanjing, Jiangsu PR China

**Keywords:** SPRY4-IT1, ZNF703, Proliferation, Breast cancer

## Abstract

**Background:**

Long noncoding RNAs (lncRNAs) have emerged recently as a new class of genes that regulate cellular processes, such as cell growth and apoptosis. The SPRY4 intronic transcript 1 (SPRY4-IT1) is a 708-bp lncRNA on chromosome 5 with a potential functional role in tumorigenesis. The clinical significance of SPRY4-IT1 and the effect of SPRY4-IT1 on cancer progression are unclear.

**Methods:**

Quantitative reverse transcriptase PCR (qRT-PCR) was performed to investigate the expression of SPRY4-IT1 in 48 breast cancer tissues and four breast cancer cell lines. Gain and loss of function approaches were used to investigate the biological role of SPRY4-IT1 in vitro. Microarray bioinformatics analysis was performed to identify the putative targets of SPRY4-IT1, which were further verified by rescue experiments, and by western blotting and qRT-PCR.

**Results:**

SPRY4-IT1 expression was significantly upregulated in 48 breast cancer tumor tissues comparedwith normal tissues. Additionally, increased SPRY4-IT1 expression was found to be associated with a larger tumor size and an advanced pathological stage in breast cancer patients. The knockdown of SPRY4-IT1 significantly suppressed proliferation and caused apoptosis of breast cancer cells in vitro. Furthermore, we discovered that ZNF703 was a target of SPRY4-IT1 and was downregulated by SPRY4-IT1 knockdown. Moreover, we provide the first demonstration that ZNF703 plays an oncogenic role in ER (−) breast carcinoma cells.

**Conclusions:**

SPRY4-IT1 is a novel prognostic biomarker and a potential therapeutic candidate for breast cancer.

**Electronic supplementary material:**

The online version of this article (doi:10.1186/s12943-015-0318-0) contains supplementary material, which is available to authorized users.

## Background

Despite the improved prognosis of breast cancer patients because of early diagnosis, radical surgery and the development of adjuvant therapy, breast cancer remains the most common type of cancer among women [1,2]. Traditional prognostic markers, such as the estrogen receptor (ER), progesterone receptor (PR), HER2/NEU, and p53 [3,4], are insufficient indicators of tumor aggressiveness and do not adequately discriminate between different biological and clinical outcomes [5]. Therefore, a better understanding of the genetic and molecular characteristics of breast cancer is urgently needed for early diagnosis, choice of the appropriate treatment and an improved prognosis for patients with breast cancer.

Recent studies have demonstrated that a class of non-protein-coding RNAs (ncRNAs), which are known as long non-coding RNAs (lncRNAs), participates in cell fate determination and human disease pathogenesis [6-9]. lncRNAs are RNA molecules longer than 200 nucleotides that are not translated into proteins [10,11]. An increasing body of evidence has suggested that lncRNAs are key regulators in several biological processes and are increasingly recognized as diagnostic or prognostic cancer biomarkers, including in breast cancer [10,12-14]. For example, GAS5, a well-known lncRNA, is markedly down-regulated in breast cancer and has been suggested as a diagnostic cancer biomarker [9,15-17]. Another characterized lncRNA, HOTAIR, is overexpressed in breast cancer, interacts with Polycomb Repressive Complex 2 (PRC2) and alters the regulation of genes [18], resulting in aberrant histone H3K27 methylation and gene expression and further promoting cancer invasiveness and metastasis [18]. Our previous studies have also shown that patients with higher lncRNA Loc554202 expression present an advanced pathological breast cancer stage [19].

lncRNA-SPRY4-IT1 (GenBank Accession ID AK024556) is derived from an intron of the SPRY4 gene. Khaitan et al. [20] observed that SPRY4-IT1 is highly expressed in melanoma cells compared with melanocytes. The knockdown of SPRY4-IT1 expression results in defects in cell growth, decreased invasion, and increased rates of apoptosis in melanoma cells [20]. A study conducted by Zou et al. [21] showed that aberrant expression of lncRNA SPRY4-IT1 may contribute to the generation of abnormal HTR-8/SVneo trophoblast cells. However, the expression profile and biological role of SPRY4-IT1 as well as its mechanism in breast cancer remain largely unknown. In this study, we assessed the effects of SPRY4-IT1 expression on breast cancer cell phenotypes in vitro. Our results suggest that increased SPRY4-IT1 expression may play a role in breast cancer carcinogenesis.

Zinc finger 703 (ZNF703) has been identified as the genetic driver of the amplification at 8p12 in luminal B tumors [22-26]. A recent study demonstrated that enhanced ZNF703 expression represses E-cadherin expression and increases lung metastases in a mouse model of breast cancer [27]. In this study, we found that ZNF703 is a downstream target gene of SPRY4-IT1 and demonstrated that ZNF703 promotes ER(−) breast carcinoma cell proliferation and suppresses apoptosis in vivo.

## Results

### Expression of SPRY4-IT1 in breast cancer tissues and breast cancer cell lines

**Figure 1:**
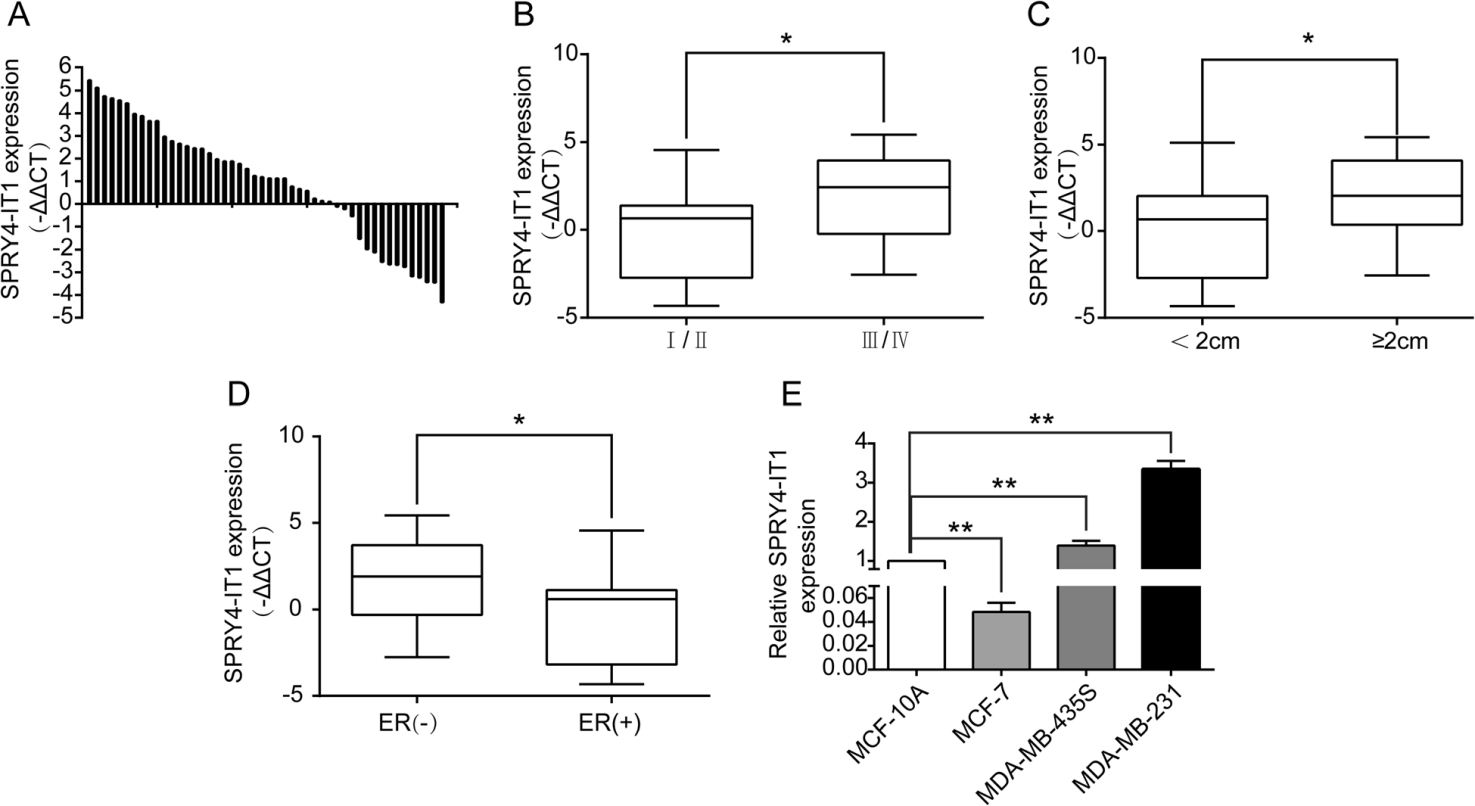
**Relative expression of SPRY4-IT1 in breast cancer tissues and cells compared with adjacent normal tissues and normal breast epithelial cells. (A)** Relative expression of SPRY4-IT1 in breast cancer tissues (T) (n = 48) compared with corresponding non-tumor tissues (N) (n = 48). SPRY4-IT1 expression was examined by qPCR and normalized to GAPDH expression and the levels in the cells of interest were compared with the expression levels inMCF-10A and other cells. The results are presented as the -ΔΔCT changes in tumor tissues relative to normal tissues. **(B and C)** The data are presented as the relative expression levels in tumor tissues. SPRY4-IT1 expression was significantly higher in patients with a higher pathological stage and a larger tumor size (shown as -ΔΔCT). **(D)** SPRY4-IT1 expression was assessed by qRT-PCR in ER(−) breast cancer tissues and ER(+) breast cancer tissues(shown as -ΔΔCT). **(E)** The relative SPRY4-IT1 expression levels in breast cancer cell lines (MD-MB-231, MDA-MB-435S and MCF-7) were compared with human breast epithelial cells (MCF-10A), as assessed by qRT-PCR. * P < 0.05 and **P < 0.01.

qRT-PCR analysis was used to examine the SPRY4-IT1 levels in 48 breast cancer tissues and 48 matched normal breast tissues. SPRY4-IT1 expression was upregulated (P < 0.05) in cancerous tissues compared with normal tissues (Figure 1A). We then evaluated the correlation of SPRY4-IT1 expression with clinicopathological parameters (i.e., stage, maximum diameter) to assess its clinical significance. As presented in Figures 1B and 1C, both larger tumors, which represent a higher tumor burden, and more advanced tumors are associated with increased SPRY4-IT1 expression. Furthermore, we found ER(−)breast cancer tissues exhibit increased SPRY4-IT1 expression than ER(+)breast cancer tissues (p < 0.01; Figure 1D). These analyses demonstrate that SPRY4-IT1 may be a potential prognostic biomarker for breast cancer patients. We then examined the expression of SPRY4-IT1 in three human breast cancer cell lines, namely MD-MB-231, MDA-MB-435S and MCF-7 cells, and in the normal breast epithelium cell line MCF-10A. Significantly higher expression of SPRY4-IT1 was found in MDA-MB-231 and MD-MB-435S cells, compared with MCF-10A cells (p < 0.01; Figure 1E), but MCF-7 cells expressed relatively lower levels of SPRY4-IT1 compared with MCF-10A cells (p <0.01; Figure 1E). These results are consistent with our previous findings. Therefore, SPRY4-IT1 was depleted in MD-MB-231 and MD-MB-435S cells, which exhibit a higher expression of SPRY4-IT1. In addition, SPRY4-IT1 was over-expressed in the MCF-7 cell line after transfection of pcDNA3.1- SPRY4-IT1, which generates a relatively lower level of SPRY4-IT1 expression. The ectopic expression and knockdown of SPRY4-IT1 in cells were confirmed by qRT-PCR (p < 0.01; Additional file 1: Figure S1A and S1B).

### SPRY4-IT1 promotes breast cancer cell proliferation in vitro

**Figure 2:**
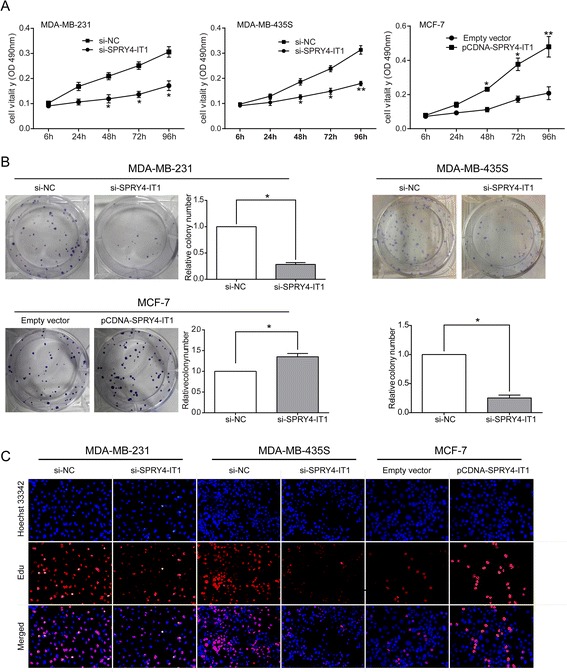
**Effects of SPRY4-IT1 on breast cancer cell proliferation in vitro. (A)** An MTT assay was performed to determine the proliferation of MDA-MB-231, MDA-MB-435S and MCF-7 cells. The data are presented as the means ± S.D. from three independent experiments. **(B)** Colony-forming growth assays were performed to determine the proliferation of MDA-MB-231, MDA-MB-435S and MCF-7 cells. The colonies were counted and captured. **(C)** The proliferating MDA-MB-231, MDA-MB-435S and MCF-7 cells were labeled with EdU. The Click-it reaction revealed EdU staining (red). The cell nuclei were stained with Hoechst 33342 (blue). The images are representative of the results obtained. *P < 0.05 and **P < 0.01.

To assess the biological role of SPRY4-IT1 in breast cancer, we observed its effect on cell proliferation. As shown in Figure 2A, MDA-MB-231 and MDA-MB-435S cells, which exhibit naturally high SPRY4-IT1 expression levels, displayed a lower cell viability rate than control cells after SPRY4-IT1 knockdown. Moreover, MCF-7 cells, which exhibit naturally low SPRY4-IT1 expression, exhibited a notably higher cell viability rate after overexpression of SPRY4-IT1 than that observed in the controls infected with the empty vector. Furthermore, cell proliferation was also measured using a colony formation assay. Compared with the control cells, SPRY4-IT1 knockdown in MDA-MB-231 and MDA-MB-435S cells resulted in markedly decreased colony formation abilities (p < 0.05; Figure 2B). Similarly, SPRY4-IT1-overexpressing MCF-7 cells displayed significantly increased colony formation (p < 0.05; Figure 2B). EdU (red)/Hoechst (blue) immunostaining also confirmed this result (Figure 2C). These findings indicate that SPRY4-IT1 may be closely associated with the proliferation of breast cancer cell lines.

### Downregulation of SPRY4-IT1 promotes G1 arrest and causes apoptosis in breast cancer cells

**Figure 3:**
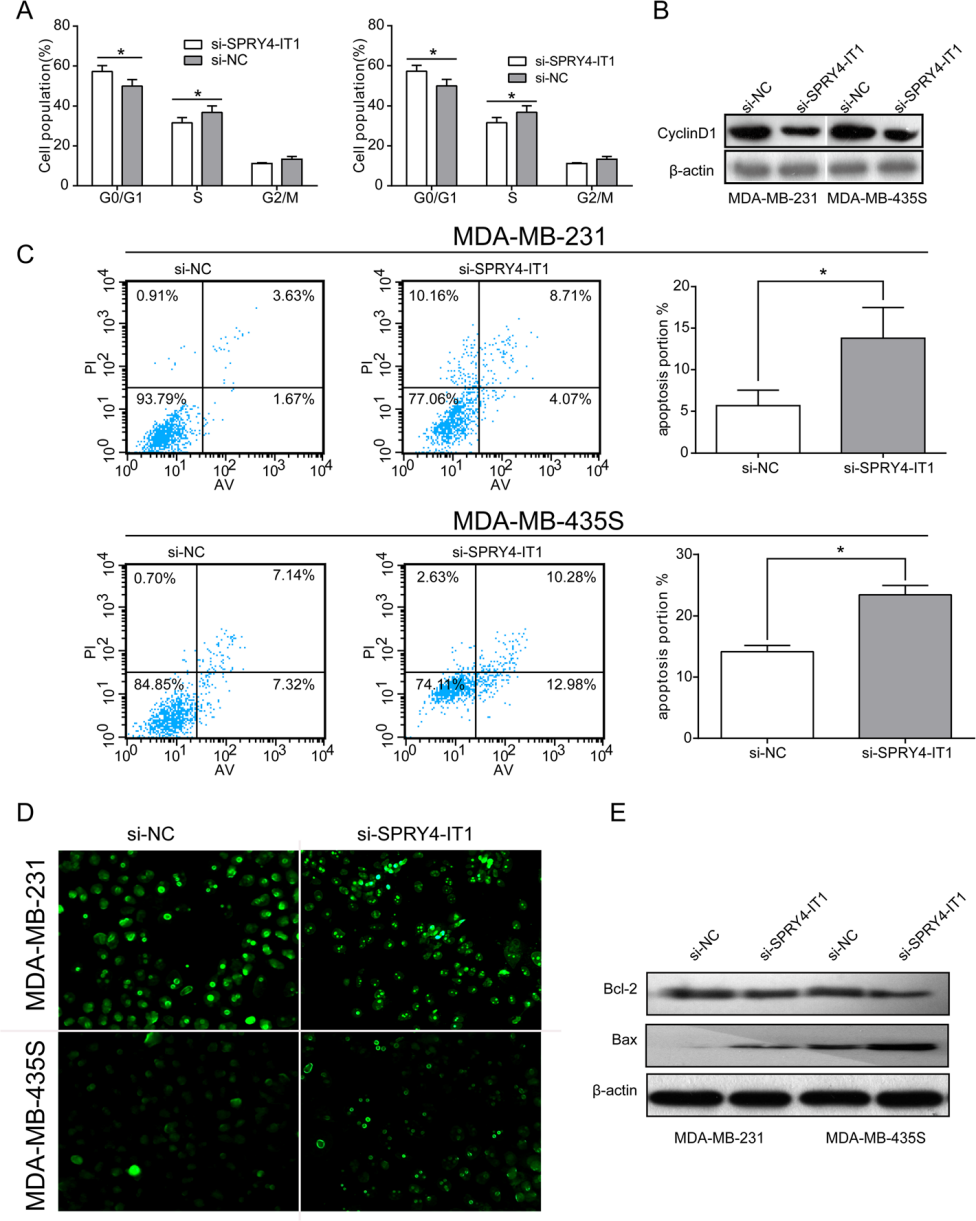
**Effect of SPRY4-IT1 on the cell cycle and apoptosis of breast cancer cells in vitro.** MDA-MB-231 and MDA-MB-435S cells were transfected with si-SPRY4-IT1 and si-NC, respectively. **(A)** The bar chart represents the percentage of cells in the G0/G1, S, or G2/M phase, as indicated. The data are presented as the means ± SD from three independent experiments. *P < 0.05. **(B)** After SPRY4-IT1 knockdown in MDA-MB-231 and MDA-MB-435S cells, the cyclinD1 protein level is diminished compared with the level observed in the control group, as determined by western blot analysis. **(C)** The percentage of apoptotic cells was determined by flow cytometric analysis. The data represent the means ± SD from three independent experiments. *P < 0.05. **(D)** Apoptosis in MDA-MB-231 and MDA-MB-435S cells after SPRY4-IT1 knockdown was detected through TUNEL staining. **(E)** The Bax and Bcl-2 protein levels were elevated in MDA-MB-231 and MDA-MB-435S cells after SPRY4-IT1 expression was blocked compared with the control group.

To probe the potential mechanisms of SPRY4-IT1 in the proliferation of breast cancer cells, we examined the cell cycle in MDA-MB-231 and MDA-MB-435S cells through flow cytometry. After treatment with si-SPRY4-IT1 or si-NC for 48 h, SPRY4-IT1 knockdown led to a significant accumulation of cells at the G0/G1-phase (p < 0.05) and a significant decrease in cells in the S-phase (p < 0.05; Figure 3A). Consistently, cyclin D1 protein expression was also disrupted in the si-SPRY4-IT1-transfected breast cancer cells compared with the control cells (Figure 3B).

**Figure 4:**
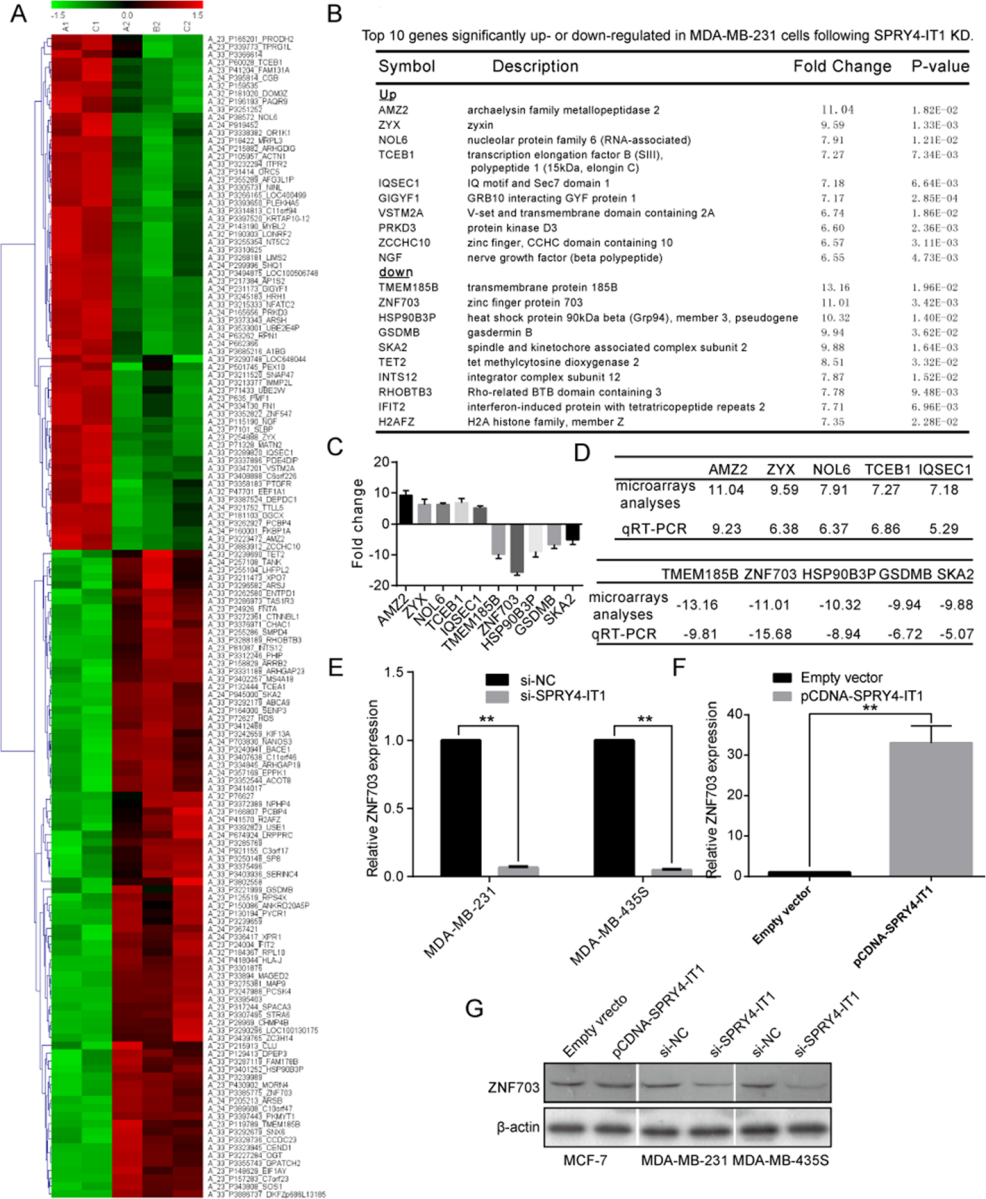
**Gene expression profiling in MDA-MB-231 cells following SPRY4-IT1 knockdown. (A)** Clusters of genes altered by SPRY4-IT1 knockdown. The heat map reveals clusters of genes. The green color indicates genes that are up regulated compared with the control cells, and the red color indicates genes that down regulated compared with the control cells. The cells in which SPRY4-IT1 was knocked down are presented as A1 and C1, and the control cells are presented as A2, B2 and C2. **(B)** Top 10 genes significantly upregulated or down regulated in MDA-MB-231 cells following SPRY4-IT1 knockdown. **(C, D)** The differential gene expression obtained from the microarray analyses was confirmed by qRT-PCR analysis of 10 selected genes using the gene specific primers shown in Additional file 2: Table S1. The data represent the means of triplicate experiments normalized to the GAPDH level and are presented as the relative fold changes (RFC) of the levels in the MDA-MB-231 cells after SPRY4-IT1 knockdown compared with the levels in the Scr-control cells. **(E, F)** qPCR analysis of the ZNF703 expression levels in MDA-MB-231 and MDA-MB-435S cells after transfection with scrambled siRNA and si-SPRY4-IT1 **(E)** and in MCF-7 cells after treatment with an empty vector and pcDNA- SPRY4-IT1 **(F)**. The data are presented as the means ± SD from three independent experiments. **P < 0.01. **(G)** The ZNF703 protein level is elevated in MDA-MB-231 and MDA-MB-435S cells after transfection with si-SPRY4-IT1 and in MCF-7 cells after transfection with pcDNA-SPRY4-IT1.

We then, investigated the effects of SPRY4-IT1 knockdown on apoptosis. The percentages of apoptotic cells were significantly increased in the treated group compared with the control group (p < 0.05; Figure 3C). Consistently, microscopic analysis of TUNEL staining in MDA-MB-231 and MDA-MB-435S cells also showed that the knockdown of SPRY40-IT1 resulted in a higher number of apoptotic cells compared with the controls (Figure 4D). Additionally, the western blot analysis show that Bax protein was significantly increased in si-SPRY4-IT1-treated cells, whereas the Bcl-2 protein level was decreased (Figure 4E). Taken together, the flow cytometric analysis, TUNEL staining and protein analysis results suggested that SPRY4-IT1 has exerts a critical effect on breast cancer cell apoptosis.

### Gene expression profiling

To analyze the transcriptional changes associated with SPRY4-IT1 knockdown, we applied microarray analysis to identify genes that exhibited a change in expression after SPRY4-IT1 knockdown in MDA-MB-231 cells. The profiling analyses identified 149 probe-set level transcripts as being significantly differentially expressed (at least a two-fold change in expression and a p-value ≤ 0.05) in cells after SPRY4-IT1 knockdown compared with control cells (Figure 4A, Additional file 2: Table S1). Of the differentially expressed genes, 66 were upregulated by at least 2-fold, and 83 were downregulated by at least 2-fold. Figure 4B summarizes the top ten upregulated or downregulated genes, organized by the average fold differences (a complete list can be found in Additional file 2: Table S1).

To validate the microarray data, qRT-PCR was used to analyze the same RNA samples subjected to transcriptome microarray analysis. Of the 14 differentially expressed genes, ten were selected: the top five upregulated genes (AMZ2, ZYX, NOL6, TCEB1, and IQSEC1) and the top five downregulated genes (TMEM185B, ZNF703, HSP90B3P, GSDMB, and SKA2). As shown in Figure 4C and 4D, the trend (upregulation or downregulation) in the change expression of all ten selected genes were consistent with the microarray analysis results, validating the accuracy of the microarray data. Moreover, we found that ZNF703 exhibited the most substantial change in gene expression in response to SPRY4-IT1 knockdown (Figure 4C and 4D). To further validate the link between SPRY4-IT1 and ZNF703 expression, we measured the ZNF703 levels in MDA-MB-231 and MDA-MB-435S cells transfected with SPRY4-IT1-siRNA and found decreased mRNA and relative protein expression levels of ZNF703, whereas MCF-7 cells transfected with pCDNA-SPRY4-IT1 exhibited increased ZNF703 mRNA and relative protein expression levels compared with the controls (Figure 4E, 4 F and 4G). Thus, ZNF703 may represent an important downstream effector of SPRY4-IT1 that potentially mediates the effects of this lncRNA on tumor growth.

### ZNF703 expression is SPRY4-IT1-inducible in vitro and is upregulated in primary breast cancer

ZNF703 has been identified as a novel oncogene in human breast cancer [22,24,25]. Recently, Reynisdottir found that high ZNF703 expression, independent of amplification, is correlated with poorer prognosis for breast cancer patients with ER-positive luminal tumors, particularly of the luminal B subtype [28]. However, it is currently unclear whether ZNF703 has similar prognostic power in ER(−) breast carcinoma.

**Figure 5:**
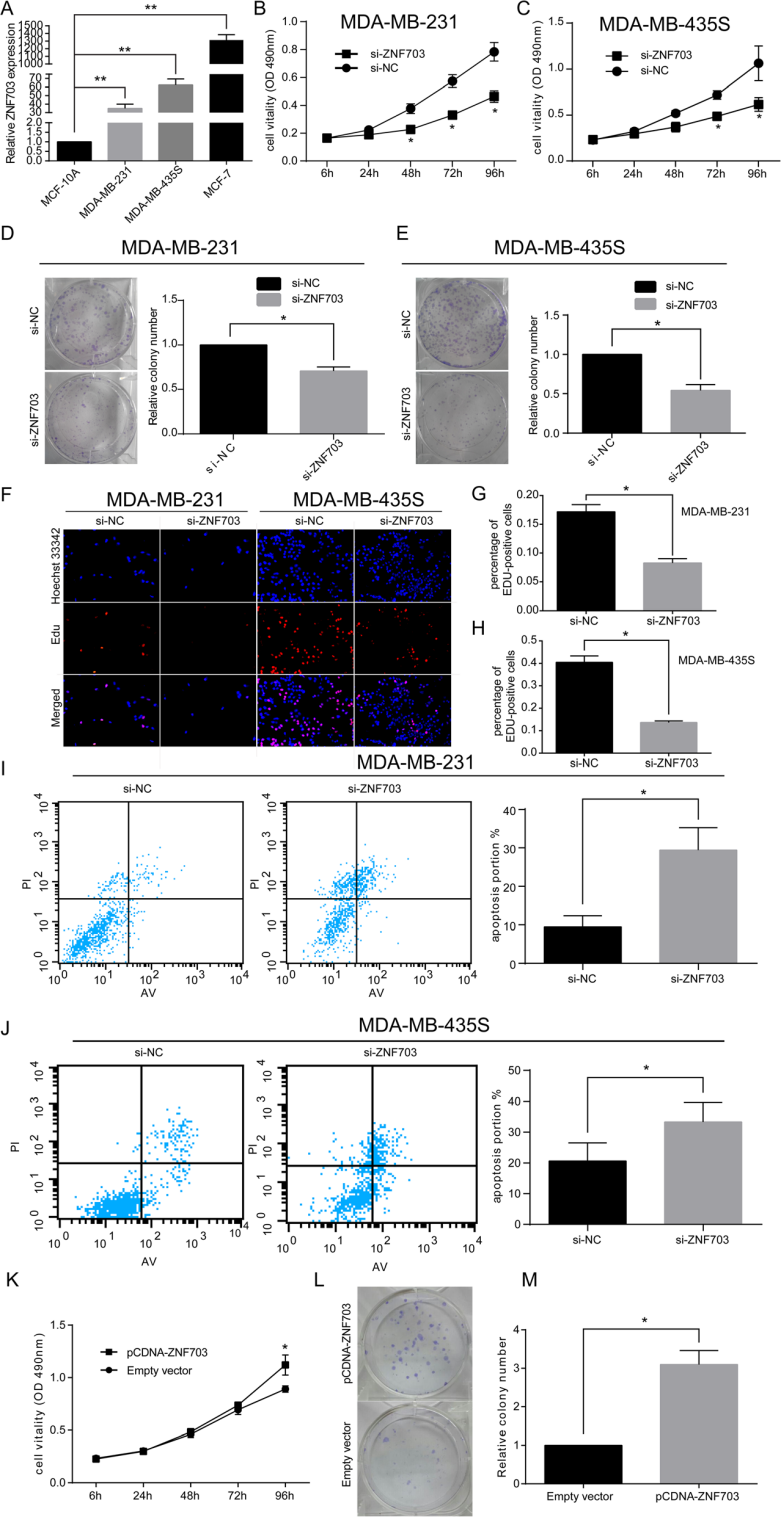
**Effects of SPRY4-IT1 on ER(−) breast cancer cell proliferation and apoptosis in vitro. (A)** The relative ZNF703 expression level in breast cancer cell lines (MDA-MB-231, MDA-MB-435S and MCF-7) compared with the level in human breast epithelial cells (MCF-10A) was assessed by qRT-PCR. **(B, C)** An MTT assay was performed to determine the proliferation of MDA-MB-231 and MDA-MB-435S cells treated with scrambled siRNA and si-ZNF703. The data are presented as the means ± S.D. from three independent experiments. **(D, E)** Colony-forming growth assays were performed to determine the proliferation of MDA-MB-231 and MDA-MB-435S cells treated with scrambled siRNA and si-ZNF703. The colonies were counted and captured. **(F, G, H)** Proliferating MDA-MB-231 and MDA-MB-435S cells treated with scrambled siRNA or si-ZNF703 were labeled with EdU. The Click-it reaction revealed EdU staining (red). The cell nuclei were stained with Hoechst 33342 (blue). The images are representative of the results obtained. **(I, J)** The percentage of apoptotic cells was determined by flow cytometric analysis. The data are presented as the means ± SD from three independent experiments. **(K)** An MTT assay was performed to determine the proliferation of MDA-MB-231 cells treated with empty vector and pcDNA-ZNF703. **(L, M)** Colony-forming growth assays were performed to determine the proliferation of MDA-MB-231 cells treated with empty vector and pcDNA-ZNF703. The colonies were counted and captured. Three independent experiments were performed for each assay. *P < 0.05 and **P < 0.01.

To assess the contribution of ZNF703 to the biological effects of ER(−) breast carcinoma, we evaluated the impact of ZNF703 silencing and overexpression on cell proliferation and apoptosis in MDA-MB-231 and MDA-MB-435S cells. First, we assessed ZNF703 expression in three human breast cancer cell lines by qRT–PCR (Figure 5A). All of the cell lines expressed notably higher levels of ZNF703 compared with human breast epithelial cells (MCF-10A), but MDA-MB-231 and MDA-MB-435S cells (ER(−)) expressed relatively lower levels of ZNF703 compared with MCF-7 cells (ER(+)).

The function of ZNF703 in ER(−) breast carcinoma cells was manipulated with small interfering RNAs (siRNAs) in MDA-MB-231 and MDA-MB-435S cells. Forty-eight hours after transfection, the ZNF703 mRNA and relative protein levels were substantially down-regulated compared with the levels observed in the respective control cells (Additional file 3: Figure S2A and S2B). Moreover, we used pCDNA-ZNF703 to up-regulate the endogenous ZNF703 expression in MDA-MB-231 cells, and the efficiency of the transfection into MDA-MB-231 was 16-fold compared with that found for the control cells (Additional file 3: Figure S2C and S2D). We then examined the impact of ZNF703 knockdown in the MDA-MB-231 and MDA-MB-435S cells lines. Compared with the control group, transfection with si-ZNF703 resulted in a significant decrease in MDA-MB-231 and MDA-MB-435S cell viability, as monitored through an MTT assay (Figure 5B and 5C). In addition, a long-term survival assay showed that ZNF703 knockdown also attenuated the colony-forming ability of the population (Figures 5D and 5E). Additionally, EdU (red)/Hoechst (blue) immunostaining also confirmed this comparison (Figures 5F, 5G and 5H).

We then investigated the effects of ZNF703 knockdown on apoptosis. As shown, the percentages of apoptotic cells were significantly increased in the treated group compared with the control group (p < 0.05; Figure 5I and 5J).

To further test the effects of ZNF703, we examined the impact of ZNF703 overexpression in the MDA-MB-231 cell line. An MTT assay was performed to monitor the effect of ZNF703 overexpression on cell growth and proliferation at different times, and a colony forming assay was also performed. The cell proliferation rate in the cultures transfected with pCDNA-ZNF703 96 h after transfection was barely distinguishable from that of the control cells (Figure 5K). Additionally, the transfection of pCDNA-ZNF703 also mildly protected the colony-forming ability of the cells compared with the negative control (Figure 5L and 5M). However, the number of apoptotic cells was not significantly decreased in the treated group compared with the control group (Additional file 4: Figure S3A and S3B). Taken together, these results suggest that ZNF703 can promote the proliferation and suppress the apoptosis of ER(−) breast cancer cells in vivo.

### SPRY4-IT1 exerts its effect through ZNF703 for both promotion of proliferation and inhibition of apoptosis

**Figure 6:**
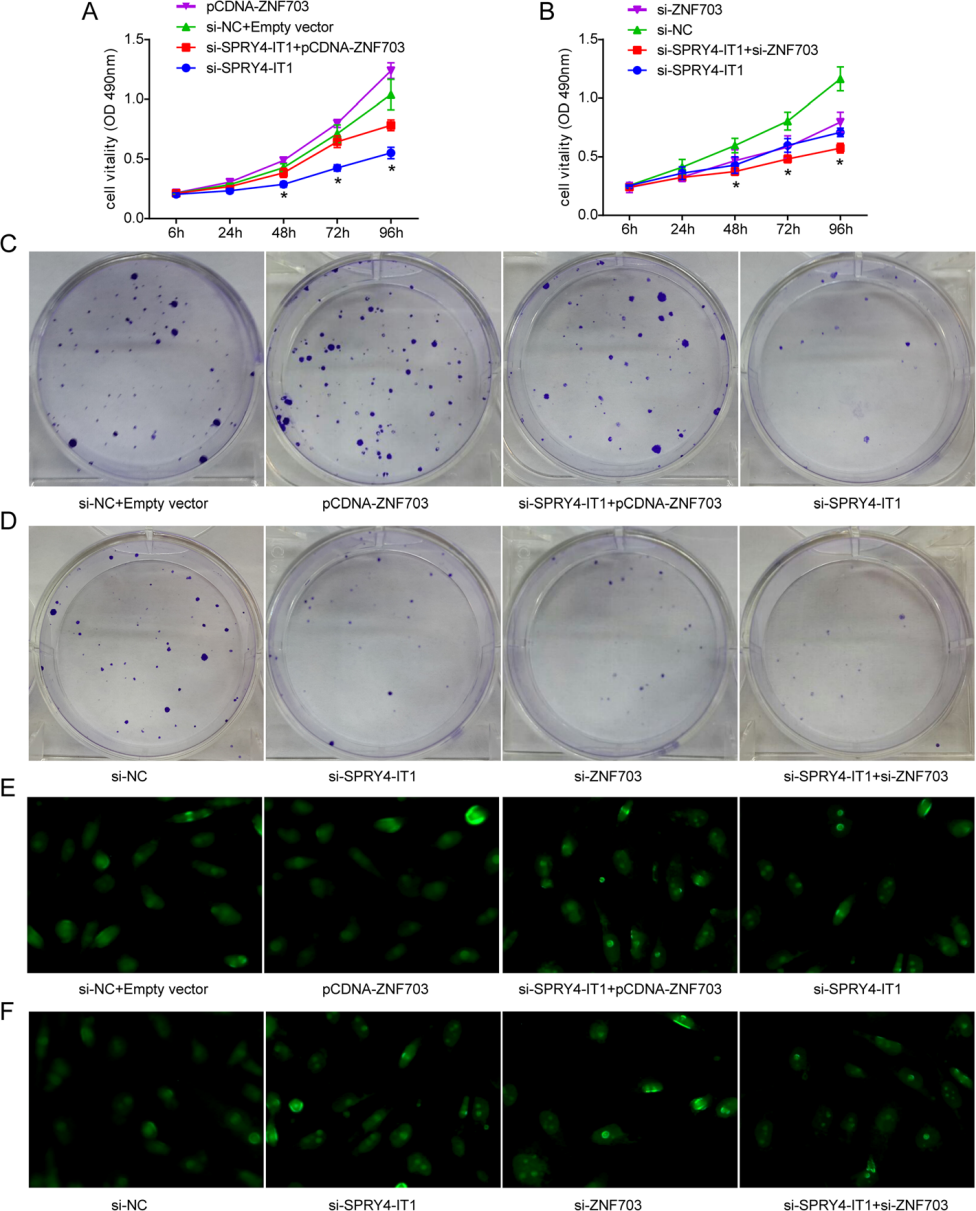
**SPRY4-IT1 promotes breast cancer cell proliferation partly through the upregulation of ZNF703 expression. (A)** An MTT assay was performed to determine the proliferation of MDA-MB-231 cells transfected with scrambled siRNA, pCDNA-ZNF703,si-SPRY4-IT1 + pCDNA-ZNF703 or si-SPRY4-IT1. **(B)** An MTT assay was performed to determine the proliferation of MDA-MB-231 cells transfected with scrambled siRNA, si-ZNF703, si-SPRY4-IT1 + si-ZNF703 or si-SPRY4-IT1. The data are presented as the means ± S.D. from three independent experiments. *P < 0.05. **(C, D)** Colony-forming growth assays were performed to determine the proliferation of MDA-MB-231 treated with scrambled siRNA, pCDNA-ZNF703, si-SPRY4-IT1 + pCDNA-ZNF703, si-ZNF703, si-SPRY4-IT1 + si-ZNF703, or si-SPRY4-IT1. **(E, F)** Apoptosis in MDA-MB-231 cells treated with scrambled siRNA, si-SPRY4-IT1 + pCDNA-ZNF703, si-SPRY4-IT1 + si-ZNF703, or si-SPRY4-IT1 was detected through TUNEL.

To investigate whether ZNF703is involved in the SPRY4-IT1induced increase in breast cancer cell proliferation, we performed rescue experiments. After transfection with si-SPRY4-IT1, MDA-MB-231 cells were cotransfected with pCDNA-ZNF703. We found that ZNF703 overexpression partially compromised the effects of SPRY4-IT1 on breast cancer proliferation (Figure 6A and 6C), whereas the knockdown of ZNF703 had the opposite effects (Figure 6B and 6D). Consistently, the TUNEL staining results also showed that ZNF703 overexpression partially compromised the effects of knockdown of SPRY40-IT1 on breast cancer apoptosis (Figure 6E), whereas the knockdown of ZNF703 had the opposite effects (Figure 6F). Our results reveal that the effect of SPRY4-IT1 on breast cancer is at least in part through targeting ZNF703.

## Discussion

It is clear that mammalian genomes encode numerous long noncoding RNAs [29-32]. Nonetheless, the functional roles of most of these transcripts remain obscure [33]. In particular, the involvement of lncRNAs in breast cancer pathogenesis and progression is not widely studied.

In our study, we first found that lncRNA SPRY4-IT1 was significantly upregulated in breast cancer tissues compared with adjacent normal tissues. Our data indicated that a high expression of this lncRNA is correlated with a larger tumor size and a later stage of tumor development in breast cancer patients, indicating that SPRY4-IT1 may be a promising prognostic biomarker for breast cancer patients. Additionally, we found that ER(−) breast cancer tissues present increased SPRY4-IT1 expression than ER(+) breast cancer tissues, which lead us to hypothesize that SPRY4-IT1 may be negatively correlated with estradiol.

Because high SPRY4-IT1 expression is associated with an aggressive tumor phenotype in breast cancer, we speculated that SPRY4-IT1 may play a significant role in tumor biology. First, we chose representative breast cancer cell lines and investigated SPRY4-IT1 expression in these cell lines comparedwith a non-tumor breast cell line. We found that the MDA-MB-231 and MDA-MB-435S cell lines exhibited high SPRY4-IT1 expression, whereas the MCF-7 cell line exhibited low SPRY4-IT1 expression compared with normal breast epithelial cells (MCF-10A), which is in agreement with our findings in breast cancer tissues. We then determined whether SPRY4-IT1 expression influences tumor-like characteristics such as proliferation and apoptosis. Indeed, the knockdown of SPRY4-IT1 inhibites cell proliferation and increases cell apoptosis in MDA-MB-231 and MDA-MB-435S cell lines, whereas the ectopic expression of SPRY4-IT1 significantly enhances cell proliferation in MCF-7 cell lines. Moreover, we demonstrated that the mechanism may be associated with G0/G1 cell cycle arrest, which is in agreement with our clinical findings that SPRY4-IT1 is significantly correlated with the tumor size and tumor stage. These results reveal that SPRY4-IT1 may affect breast cancer progression by affecting cell proliferation and apoptosis.

Although SPRY4-IT1 has been shown to play crucial biological roles and is dysregulated in various human cancers [20,21,34,35], the precise regulatory mechanisms of SPRY4-IT1 expression remain largely unknown. A previous study demonstrated that the Polycomb group protein enhancer of zeste homolog 2 (EZH2) can regulate the transcript levels of SPRY4-IT [34]. However, the downstream pathway is not currently known. To explore the molecular mechanism through which SPRY4-IT contributes to cell proliferation and causes apoptosis in breast cancer cells, we investigated potential target genes involved in cell proliferation and apoptosis through a microarray analysis. We found that the ZNF703 exhibits the most substantial expression change in response to SPRY4-IT1 knockdown. Moreover, qRT-PCR analysis demonstrated that the ZNF703 mRNA levels are reduced or elevated after the knockdown or overexpression of SPRY4-IT1, respectively. The western blot analysis also confirmed that the ZNF703 protein levels are regulated by SPRY4-IT1.

ZNF703, a gene that plays an oncogenic role in luminal B type breast cancer [22-24], is likely the most functionally important gene in the 8p12 amplicon [22,24-26]. Its role in breast cancer progression or metastasis was demonstrated in a mouse model of breast cancer [27]. These functions suggest that ZNF703 plays an important role in breast cancer formation and progression [28]. However, few studies have examined the biological function of ZNF703 in ER (−) breast carcinoma. In this study, we provide the first demonstration that ZNF703plays an oncogenic role in ER (−) breast carcinoma cells and that the expression level of ZNF703 is higher in MDA-MB-231 and MDA-MB-435S cells compared with human breast epithelial cells (MCF-10A). The knockdown of ZNF703 was found to inhibit cell proliferation and increase cell apoptosis in MDA-MB-231 and MDA-MB-435S cell lines, whereas the ectopic expression of ZNF703 significantly enhancs cell proliferation in MCF-7 cell lines. Moreover, we demonstrate that the mechanism may be associated with G0/G1 cell cycle arrest. To further confirm that ZNF703 is involved in the SPRY4-IT1-induced increase in breast cancer cell proliferation, we performed rescue experiments. The results showed that the cotransfection of si-SPRY4-IT1 and pCDNA-ZNF703 partially rescued the proliferation induced by SPRY4-IT1 upregulation and the apoptosis induced by SPRY4-IT1 downregulation, indicating that SPRY4-IT1 increases cell proliferation and inhibits cell apoptosis partially through ZNF703. However, the precise molecular mechanism regulating how SPRY4-IT1 controls ZNF703 remains unclear and requires further investigation.

## Conclusion

In summary, this study shows that the expression of the lncRNA SPRY4-IT1 is increased in breast cancer tissues and that increased expression of SPRY4-IT1 is significantly associated with a larger tumor size and a later stage of tumor development in breast cancer patients. Moreover, the knockdown of SPRY4-IT1 has the effects of suppressing breast cancer cell proliferation and causing apoptosis. Further insights into the functional and clinical implications of SPRY4-IT1 and its target ZNF703 may facilitate the identification of novel diagnostic or predictive biomarkers and drug targets for breast cancer.

## Materials and methods

### Tissue collection

Paired breast cancer and adjacent normal breast tissue were obtained from 48 patients who had undergone surgical breast cancer resection between 2012 and 2013 at Second Affiliated Hospital of Nanjing Medical, China. Local or systemic treatment had not been performed in these patients prior to the operation, and the clinicopathological characteristics of the patients with breast cancer are summarized in Additional file 5: Table S2. Samples were immediately macrodissected at the time of surgery and placed directly in RNA*Later* stabilization solution (Qiagen, Hilden, Germany). All of the tissues were stored at −80°C until total RNA was extracted. The ER status, pathological stage, grade and nodal status were appraised by an experienced pathologist. Clinicopathological characteristics including tumor-node-metastasis (TNM) staging were also scored. The non-tumorous tissues were 5 cm from the edge of the tumor, contained no obvious tumor cells and were also evaluated by the pathologist. All of the experiments were approved by the Research Ethics Committee of the Second Affiliated Hospital of Nanjing Medical University and written informed consent was obtained from all patients.

### Cell lines and culture conditions

The human breast cancer cell lines MD-MB-231 MD-MB-435S MCF-10A and MCF-7 were purchased from the Institute of Biochemistry and Cell Biology of the Chinese Academy of Sciences (Shanghai, China). MD-MB-231 and MD-MB-435S were cultured in Leibovitz’s L-15 Medium (L-15; Gibco) in humidified air at 37°C with 100% air. MCF-10A and MCF-7 were cultured in Dulbecco’s Modified Eagle’s Medium (DMEM; Invitrogen) in humidified air at 37°C with 5% CO2. All of the media were supplemented with 10% fetal bovine serum (10% FBS), 100U/ml penicillin, and 100 mg/ml streptomycin (Invitrogen, Shanghai, China).

### RNA extraction and qRT-PCR analyses

RNA extraction and qRT-PCR analyses were performed as described previously [19]. The primer sequences are shown in Additional file 6: Table S3.

### Western blot assay and antibodies

Western blot analysis was performed as previously described [19]. **β**-actin was used as a loading control, and the mean ± SD was calculated from 3 individual experiments. **β**-actin (1:1,000) antibody was used as a control and purchased from Sigma-Aldrich (USA). Anti-cyclinD1, anti-bcl-2, and anti-bax (1:1,000) antibodies were purchased from Cell Signaling Technology, Inc. (CST). The anti-ZNF703 (1:1,000) antibody was purchased from Abcam (USA).

### Small interfering RNA and plasmids DNA transfections

Small interfering RNA (siRNA) and nonspecific control siRNA was synthesized (Carlsbad, California, USA) and transfected using Lipofectamine 2000. The sequences of the siRNAs are described in Additional file 6: Table S3. The SPRY4-IT1 and ZNF703 sequences were synthesized and subcloned into the pCDNA3.1 (Invitrogen, Shanghai, China) vector. The pCDNA constructs or the empty vector were transfected into breast cancer cells cultured on six-well plates according to the manufacturer’s instructions. The empty vector was used as the control. The expression level of SPRY4-IT1 and ZNF703 was detected by qRT-PCR.

### Determination of cell viability and colony formation assay

Forty-eight hours after siRNA or DNA transfection, 3000 cells per well were seeded into 96-well plates. After 6, 24, 48, 72 and 96 h of culture, cell viability was measured using the Cell Proliferation Reagent Kit I (MTT; Roche Applied Science) as described previously [19].

Clonogenic assays were performed as described previously [19]. The colony formation ratio was calculated as “number of cells/initiative cell × 100 (%)”.

### Cell apoptosis and cell cycle analysis

Cell apoptosis was analyzed 48 h after transfection by Annexin V and propidium iodide (PI) staining as described previously [19]. Cell cycle analysis was performed 48 h after transfection with PI staining as described previously [19]. Three independent experiments were performed for each assay.

### Ethynyl deoxyuridine (Edu) analysis

Proliferating cells were assessed using the 5-ethynyl-2-deoxyuridine (EdU) labeling/detection kit (Ribobio, Guangzhou, China) according to the manufacturer’s protocol. Briefly, breast cancer cells were cultured in 96-well plates at 5 × 10^3^ cells per well and transfected with plasmid DNA or siRNA for 48 h. Then, 50 μM EdU labeling medium was added to the cell culture and incubated for 2 h at 37°C under 5% CO2. Next, the cultured cells were fixed with 4% paraformaldehyde (pH 7.4) for 30 min and treated with 0.5% Triton X-100 for 20 min at room temperature. After washing with PBS, the samples were stained with anti-EdU working solution at room temperature for 30 min. Subsequently, the cells were incubated with 100 μL Hoechst 33342 (5 μg/mL) at room temperature for 30 min, followed by observation under a fluorescent microscope. The percentage of EdU-positive cells was calculated from five random fields in three wells.

### TUNEL staining

Terminal deoxynucleotidyl transferase-mediated dUTP nick end labeling (TUNEL) was performed with an apoptosis detection kit (KeyGEN BioTECH, China) accordingly to the manufacturer’s instructions. Randomly selected field without significant necrosis in 10 high-power fields (6400) were assessed for TUNEL-positive cells. The index of TUNEL was calculated based on the number of total nuclei and cells with green nuclei.

### Microarray bioinformatics analysis

Total RNA, cRNA probe preparation, array hybridization and data analysis were performed as described in the Affymetrix manufacturer’s instructions. AffymetrixTM HG-U133 Plus 2.0 whole genome chips were used (Santa Clara, CA, USA). RNA log expression units were calculated from the Affymetrix GeneChip array data using the GeneSpring GX (Agilent, Santa Clara, CA, USA). The default settings were used to background correct, normalize and summarize all expression values. Significant differences between sample groups were identified using the *t*-test based on a normalized value for each gene, and a *p*-value was calculated using a modified permutation test. Heatmap and principle component analysis (PCA) were performed using GeneSpring GX software (Agilent, Santa Clara, CA, USA) to provide a visual representation of how the various sample groups are related. Gene annotation and gene ontology were performed with Amigo. The microarray data has been submitted to Gene Expression Omnibus (GEO) database (GSE62507).

### Statistical analysis

Statistical analysis was performed using SPSS software (SPSS, Inc., Chicago, IL, USA). Clinicopathological data were analyzed using the chi-square exact test. For comparing the SPRY4-IT1 expression in breast cancer tissues and matched normal breast tissues, paired *t*-test was used. For comparisons between two samples, an unpaired two-tailed *t*-test was performed. A *p*-value of <0.05 was considered statistically significant. The results are reported as the means ± SDs. Statistical significance was assigned at P < 0.05 (*) or P < 0.01 (**). All experiments were performed at least three times with triplicate samples.

## Additional files

Additional file 1: Figure S1. qPCR analysis of the SPRY4-IT1expression levels in MDA-MB-231 and MD-MB-435S cells after transfection with scrambled siRNA and si- SPRY4-IT1 and in MCF-7 cells after treatment with empty vector and pcDNA-SPRY4-IT1. All of the experiments were performed in triplicate and the data are presented as the means ± SD. *P < 0.05 and **P < 0.01. Additional file 2: Table S1. The profiling analyses identified probe-set level transcripts as being significantly differentially expressed in cells after SPRY4-IT1 knockdown compared with control cells. Additional file 3: Figure S2. qPCR and western blot analysis of the ZNF703 expression levels in MDA-MB-231 and MD-MB-435S cells after treatment with scrambled siRNA and si- ZNF703 and in MCF-7 cells after treatment with empty vector and pcDNA-ZNF703. All of the experiments were performed in triplicate and the data are presented as the means ± SD. **P < 0.01. Additional file 4: Figure S3. The percentage of apoptotic cells was determined by flow cytometric analysis. The data are presented as the means ± SD from three independent experiments. Additional file 5: Table S2. The clinical characteristics of the BC Patients. Additional file 6: Table S3. Sequence of primers and siRNA.

## Abbreviations

lncRNAs: Long noncoding RNAs
SPRY4-IT1: SPRY4 intronic transcript 1
qRT-PCR: Quantitative reverse transcriptase Polymerase Chain Reaction
ER: Estrogen receptor
PR: Progesterone receptor
HER2/neu: Human epidermal growth factor receptor-2
ncRNAs: Non-protein-coding RNAs
RNA: Ribonucleic Acid
GAS5: Growth arrest-specific transcript 5
HOTAIR: HOX transcript antisense RNA
PRC2: Polycomb Repressive Complex 2
ZNF703: Zinc finger 703
TNM: Tumor-node-metastasis
DMEM: Dulbecco’s Modified Eagle’s Medium
FBS: Fetal bovine serum
siRNA: Small interfering RNA
MTT: 3-(4,5-Dimethylthiazol-2-yl)-2,5-diphenyltetrazolium bromide
PI: Propidium iodide
Edu: Ethynyl deoxyuridine
PBS: Phosphate buffer saline
TUNEL: Terminal deoxynucleotidyl transferase-mediated dUTP nick end labeling
EZH2: Enhancer of zeste homolog 2
