# Supplementary figures and images for: The long noncoding RNA SPRY4-IT1 increases the proliferation of human breast cancer cells by upregulating ZNF703 expression
Source: Mol Cancer. 2015 Feb 22;14:51. doi: 10.1186/s12943-015-0318-0 (PMC4350857; doi:10.1186/s12943-015-0318-0)

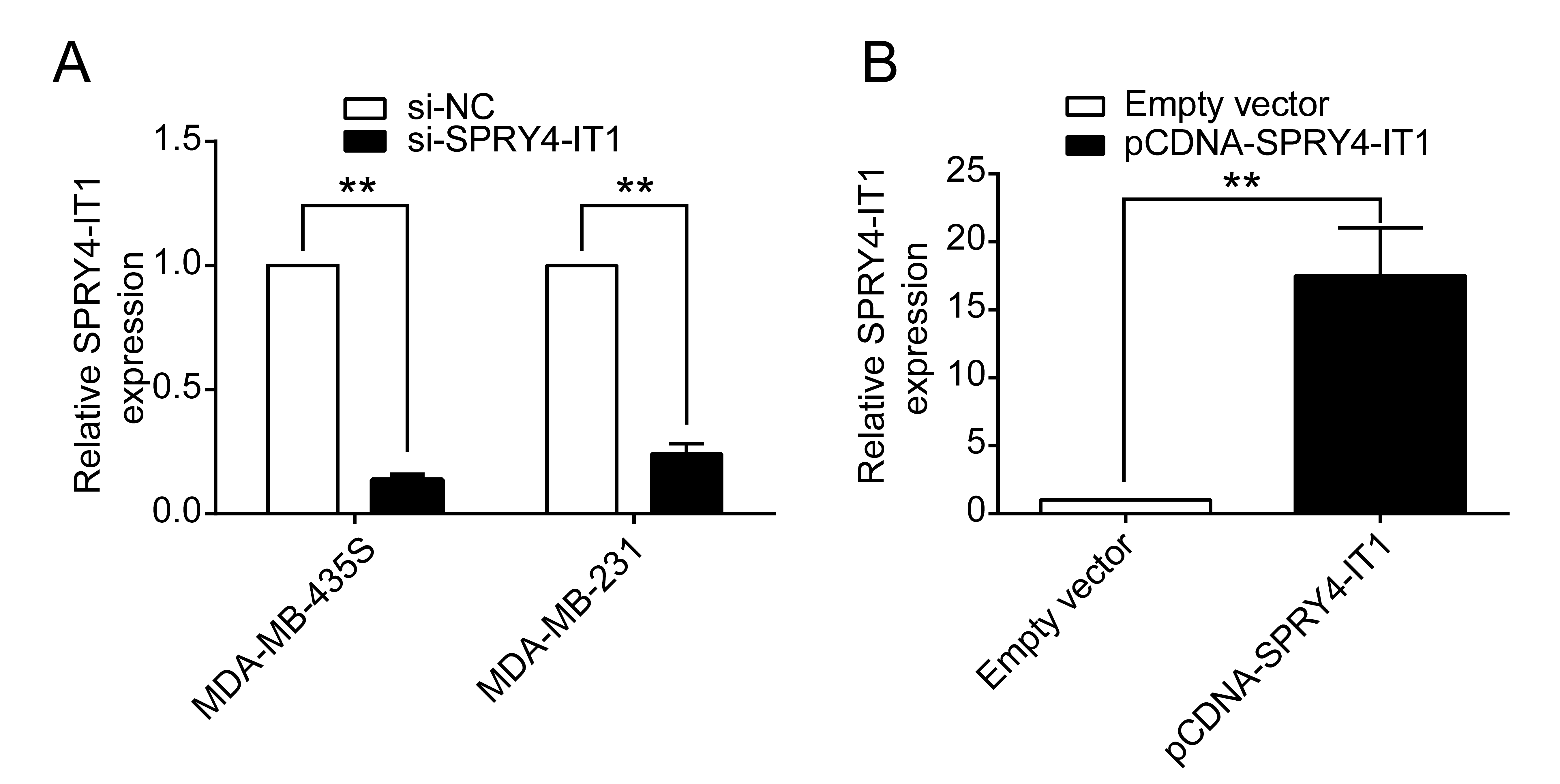

Supplement: Additional file 1: Figure S1. — qPCR analysis of the SPRY4-IT1expression levels in MDA-MB-231 and MD-MB-435S cells after transfection with scrambled siRNA and si- SPRY4-IT1 and in MCF-7 cells after treatment with empty vector and pcDNA-SPRY4-IT1. All of the experiments were performed in triplicate and the data are presented as the means ± SD. *P < 0.05 and **P < 0.01. [file 12943_2015_318_MOESM1_ESM.tiff]

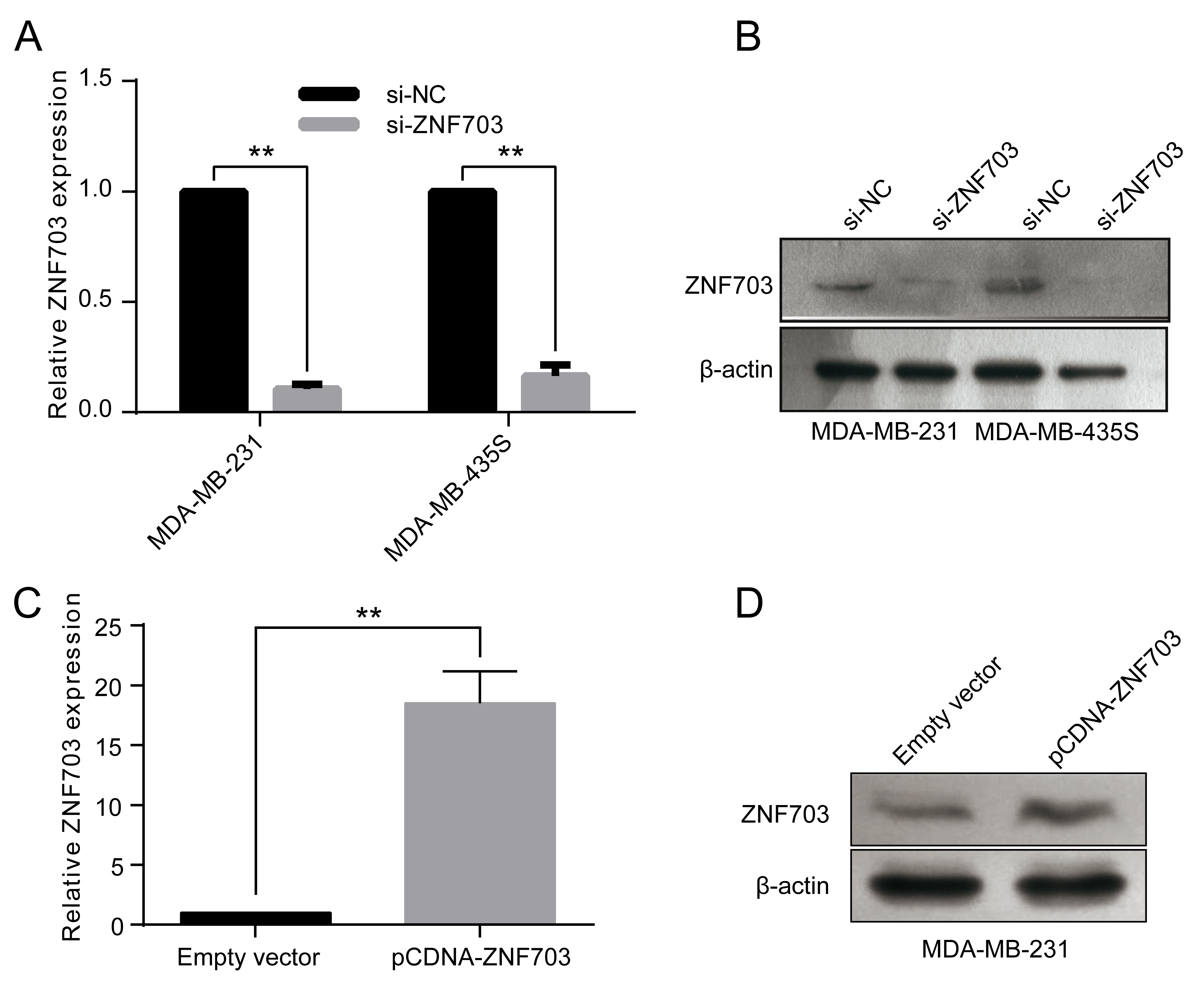

Supplement: Additional file 3: Figure S2. — qPCR and western blot analysis of the ZNF703 expression levels in MDA-MB-231 and MD-MB-435S cells after treatment with scrambled siRNA and si- ZNF703 and in MCF-7 cells after treatment with empty vector and pcDNA-ZNF703. All of the experiments were performed in triplicate and the data are presented as the means ± SD. **P < 0.01. [file 12943_2015_318_MOESM3_ESM.tiff]

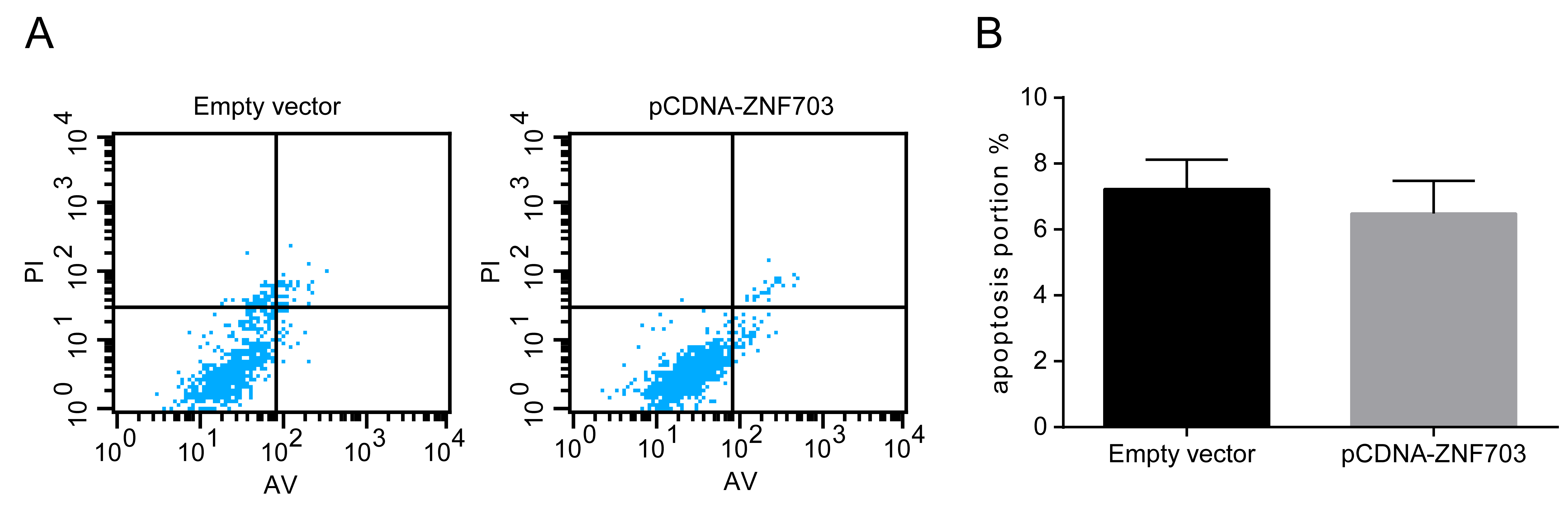

Supplement: Additional file 4: Figure S3. — The percentage of apoptotic cells was determined by flow cytometric analysis. The data are presented as the means ± SD from three independent experiments. [file 12943_2015_318_MOESM4_ESM.tiff]
