# Supplementary material for: The long noncoding RNA SPRY4-IT1 increases the proliferation of human breast cancer cells by upregulating ZNF703 expression
Source: Mol Cancer. 2015 Feb 22;14:51. doi: 10.1186/s12943-015-0318-0 (PMC4350857; doi:10.1186/s12943-015-0318-0)
Supplement: Additional file 5: Table S2. — The clinical characteristics of the BC Patients. [file 12943_2015_318_MOESM5_ESM.pdf]

Table 1. The Clinical Characteristics of the BC Patients

| Variables | No. of cases | Relative SPRY4-IT1 expression | | |
|---|---|---|---|---|
| | | Low | High | P-value[a] |
| Age(years) | | | | 0.241 |
| ＜45 | 22 | 5 | 17 | |
| ≥45 | 26 | 10 | 16 | |
| Maximum tumor size | | | | 0.015* |
| ＜2cm | 26 | 12 | 14 | |
| ≥2cm | 22 | 3 | 19 | |
| ER status | | | | 0.005** |
| Positive | 18 | 10 | 8 | |
| Negative | 30 | 5 | 25 | |
| Differentiation | | | | 0.272 |
| Well, moderate | 17 | 7 | 10 | |
| Poor | 31 | 8 | 23 | |
| No. of positive nodes | | | | 0.091 |
| ＜4 | 30 | 12 | 18 | |
| ≥4 | 18 | 3 | 15 | |
| Tumor stage | | | | 0.017* |
| Ⅰ/Ⅱ | 23 | 11 | 12 | |
| Ⅲ/Ⅳ | 25 | 4 | 21 | |

[a] Chi-squared test. * P<0.05 ** P<0.01
