# Supplementary material for: The long noncoding RNA SPRY4-IT1 increases the proliferation of human breast cancer cells by upregulating ZNF703 expression
Source: Mol Cancer. 2015 Feb 22;14:51. doi: 10.1186/s12943-015-0318-0 (PMC4350857; doi:10.1186/s12943-015-0318-0)
Supplement: Additional file 6: Table S3. — Sequence of primers and siRNA. [file 12943_2015_318_MOESM6_ESM.pdf]

Table 1 Sequence of primers and siRNA

Gene name	Forward（5'--3'）	Reverse（5'--3'）
AMZ2	ACACCCTCTCCAAACAAACG	ACAGTAGCCCGTGAGCCATT
ZYX	CACCTCCTTCATCGTGGAC	ACTCGCACAGTCTCATCTCG
NOL6	TCGGATTGATGCCTTCCTAC	GCTGCCCACAACAGTAACCT
TCEB1	TCGCCTCACCAGGAAACTAC	CCTTCACAGCCACCATAGG
IQSEC1	CTCCACTTCTTGCCTGAGC	AAGCCATCCTGTGTGAATCC
TMEM185B	GCCTGTGTGGAGTTCAAAGC	AAGACCAGCAGCCAGAAGTG
HSP90B3P	TTGAGAAGGCTGTGGTGTCT	TTGCTCGTTTGGTATGCTTG
GSDMB	GTCTTTGGGTTCGGAGGATT	GCGAGGGAGTTTAGCACATC
SKA2	TGGGAAATGCTGAAACTATGC	TCCAAACATCCTGACACTCAA
ZNF703	CCCTCCAGCATTGGCTACC	CAATAGGGGTCGCGGCATAAT
GAPDH	GACTCATGACCACAGTCCATGC	AGAGGCAGGGATGATGTTCTG
SPRY4-IT1	TAAGCTTGTAGAGATGGGGGTTTCATCCTGTTGG	ACTCGAGAAAGACTCCCTTTCCTTAAGCAGATTCAC
si-SPRY4-IT1	CCCAGAATGTTGACAGCTGCCTCTT	
si-ZNF703	CCACACACUUUGGGCCUAA	
